# The influence of pre-heating the restoration and luting agent on the flexural strength of indirect ceramic and composite restorations

**DOI:** 10.1080/26415275.2023.2279066

**Published:** 2023-11-16

**Authors:** João Paulo Mendes Tribst, Lilis Etoeharnowo, Maril Tadros, Albert J. Feilzer, Arie Werner, Cornelis J. Kleverlaan, Amanda Maria de Oliveira Dal Piva

**Affiliations:** a Department of Reconstructive Oral Care, Academic Centre for Dentistry Amsterdam (ACTA), Universiteit van Amsterdam and Vrije Universiteit, Amsterdam, North Holland, the Netherlands; b Department of Dental Materials Science, Academic Centre for Dentistry Amsterdam (ACTA), Universiteit van Amsterdam and Vrije Universiteit, Amsterdam, North Holland, the Netherlands

**Keywords:** Dental Materials, Finite Element Analysis, Luting Cements, Pre-heated Resin Composites, Resin Composites

## Abstract

**Background:**

This study investigated the impact of luting procedure and restoration thicknesses on the flexural strength of CAD/CAM restorations. Traditional luting agents have been questioned in favor of pre-heated resin composites or flowable composites.

**Materials and Methods:**

400 disc-shaped restorations (lithium disilicate [IPS e.max CAD] or resin composite [Tetric CAD, Ivoclar]) were cemented onto dentin analog discs using different procedures (n = 20): dual-curing resin cement (Panavia V5), light-curing resin cement (Panavia Veneer LC), pre-heated resin composite (Clearfil™ AP-X) with or without pre-heated restoration, and high-filled flowable composite (Clearfil Majesty™ Flow). The biaxial flexural strength was calculated.

**Results:**

There were significant effects of material, thickness, and luting procedure on flexural strength (p < 0.001). Resin composite specimens exhibited lower flexural strength (90 MPa) compared to lithium disilicate specimens (571 MPa), with thicker restorations (338 MPa) being stronger than thinner ones (323 MPa). Light-curing cement showed the highest strength (408.8 MPa)^A^, followed by dual-curing cement (362 MPa)^B^, pre-heated cement with pre-heated composite (318 MPa)^C^, pre-heated composite (304 MPa)^C^, and flowable resin composite (259 MPa)^D^. The light-curing cement yielded similar results to the pre-heated resin composite associated or not with the pre-heated crown for the thicker lithium disilicate specimens, whereas for the thinner lithium disilicate specimens all luting procedures performed similarly. Thin resin composite discs showed higher flexural strength when luted with light-curing cement, whereas the luting procedure had less influence for the thicker restorations.

**Conclusion:**

Luting procedures impact the flexural strength of CAD/CAM lithium disilicate and resin composite restorations. Pre-heated resin composite, with or without pre-heated restoration, can replace dual-curing cement. Nevertheless, light-curing cement is superior for resin composite and 1.5 mm lithium disilicate restorations.

## Key-messages

1. Different luting procedures significantly impact the flexural strength of CAD/CAM lithium disilicate and resin composite restorations, with light-curing cement demonstrating superior performance for specific thicknesses.
2. Pre-heated resin composites, either with or without pre-heated restorations, offer a viable alternative to conventional dual-curing cement for bonding indirect restorations, presenting potential clinical advantages.
3. Restoration thickness plays a crucial role in the mechanical response of restorations, with thinner resin composite restorations benefiting from dual- or light-curing, while thicker ones are less sensitive to luting procedure variations.

## Introduction

Adhesive dentistry allowed the development of minimal invasive preparations to restore damaged teeth. Therefore, partial indirect restorations, e.g., onlays, inlays, and overlays, are considered reliable alternatives to restore function and anatomy. To cement these restorations, self-adhesive, and conventional resin cements have been widely employed to achieve durable performance associated with easy handling due to their adequate viscosity. In addition, for successful luting, the resin cement must provide enough bond- and cohesive strength, esthetics, and marginal sealing, and contribute to the color stability (Sousa et al., 2020). The light-curing resin cements are indicated to bond translucent restorations with thicknesses below 2 mm. However, dual-curing resin cements share some similar indications and can be used when the intensity of the curing light is compromised due to the thickness or low translucency of the restoration. In such cases, the chemical reaction can initiate after the light-curing activation, ensuring complete polymerization after a certain period. Hence, dual-curing cements provide a more versatile option for clinicians with reduced working time.

Despite the availability of resin cement, there has been advocacy for using heated resin composites in cementing indirect restorations. This is because resin composites possess a higher filler content compared to resin cement. By heating the resin composites, their flowability is enhanced, making it easier to remove excess material and achieve better adaptation when used as a luting agent (Rocca, 2007; El-Deeb et al., 2015; Connor and Gavriil, 2021). The clinical protocol typically involves placing the resin composite in a heating device (Rueggeberg et al., 2010; El-Deeb et al., 2015; Reboul et al., 2018) or an oven (Lohbauer et al., 2009; Tomaselli et al., 2019; Gugelmin et al., 2020). However, there is currently limited data in the literature that confirms the influence of filler content on the mechanical strength of cemented restorations. Some limitations have also been reported for the heating resin composite technique: these include the restricted working time until the viscosity of the resin composite increases again, the need for meticulous pressure during restoration insertion, extended light curing, and high polymerization shrinkage (Connor and Gavriil, 2021). In addition, there is no available data on the association of heated composite with a heated restoration to reduce the temperature mismatch between the two during the clinical procedure.

Dentists who wish to avoid the heating procedure can use high-filled flowable resin composites for bonding purposes (Furuse at al., 2015, Mutlu et al., 2021). However, it is important to note that there is currently a lack of investigation regarding these specific luting procedures for indirect ceramic and composite restorations. Furthermore, research is needed to evaluate the efficacy and suitability of these high-filled flowable resin composites in various clinical scenarios. Therefore, one of the main questions remains: which protocol promotes a stronger restoration when cementing CAD/CAM resin composite or lithium disilicate restorations using dual-curing resin cement, light-curing resin cement, heated composite, heated composite, and heated restoration, or high-filled flowable composite?

To evaluate the flexural strength of a tri-layer bonded restoration, the load-to-failure force can be used with finite element analysis to determine the biaxial flexural strength according to the center of maximum tensile stress (Tribst et al., 2020). Therefore, this study aimed to investigate the influence of luting procedures and ceramic thickness on the load-to-failure and the biaxial flexural strength of cemented lithium disilicate or resin composite CAD/CAM restorations. The hypotheses were 1) Different indirect materials would promote different flexural strengths when bonded to a dentin analog; 2) The restoration thickness would affect the restoration flexural strength; 3) Different luting procedures would promote different flexural strength values for both bonded indirect materials in different thicknesses.

## Materials and Methods

**Figure 1.**
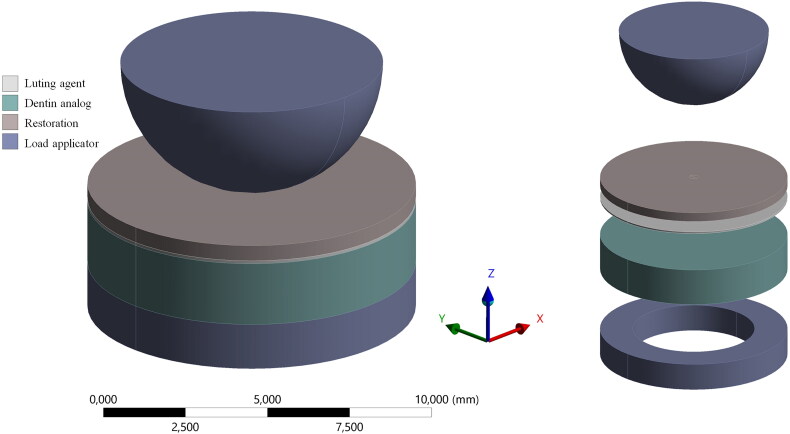
Schematic representation of the setup of the tri-layer biaxial flexural test method, with loading and support ring used for the experiment and FEA calculations.

Four hundred tri-layer specimens (Tribst et al., 2019, Guilardi et al., 2019) consisting of lithium disilicate (IPS e.max CAD) or resin composite (Tetric CAD), different luting agents, and standardized epoxy resin discs were constructed and subjected to biaxial flexural test method to determine the initial load to failure (see Figure 1). The detailed procedure is described below.

### Specimen preparation

Blocks of lithium disilicate (LD - IPS e.max CAD, Ivoclar, Schaan, Lichtenstein. Batch numbers: Z03B1G and Z034M8) and resin composite for CAD/CAM (RC - Tetric CAD, Ivoclar. Batch numbers: Z030F7, Z03KMK, and Z03P53) were shaped into cylinders (Ø = 10 mm) (Ferrario et al., 1999) and sliced into discs with thickness of 0.5 or 1.5 mm (n = 200, N = 400). The thicknesses were intended to simulate respectively veneers and crowns. In sequence, one side of the discs was considered as a bonding surface and was standardized with polishing using #600 and #1200 silicon-carbide papers. To simulate dentin, epoxy resin discs with a standardized thickness of 2 mm and a diameter of 10 mm were used. Before cementing the specimens, all discs were ultrasonically cleaned in isopropyl alcohol for 5 min (Tribst et al., 2019). Lithium disilicate discs were crystallized according to the manufacturer’s instructions. Table 1 presents the materials used.

**Table 1.** Materials used in the study.

Material	Brand Name, manufacturer, and batch number	Composition
Lithium disilicate	IPS® e.max CAD, Ivoclar. Batch number: Z03B1G and Z034M8	SiO_2_, Li_2_O, K_2_O, P_2_O_5_, ZrO_2_, ZnO, Al_2_O_3_, MgO, colouring oxides
Resin composite	Tetric CAD, Ivoclar. Batch numbers: Z030F7, Z03KMK, and Z03P53.	Monomers: Bis-GMA,UDMA,Bis-EMA, TEGDMA. And 71% filler weight: barium glass (< 1 µm) and silicon dioxide (<20 nm)
Dentin analog	G10/FR-4, CARBOTEC. Batch number: 295787	Glass fiber, epoxy resin
<5% hydrofluoric acid	IPS® Ceramic Etching Gel, Ivoclar. Batch number: Y48112 and Z037BV	Hydrofluoric Acid
<10% buffered hydrofluoric acid	Ultradent® Porcelain Etch, Ultradent. Batch number: BKSS4	Hydrofluoric Acid
Dentin primer	Panavia™ V5 Tooth Primer, Kuraray. Batch number: 210105	MDP, HEMA, hydrophilic aliphatic dimethacrylate, accelerators, water
Silane-based coupling agent	Clearfil™ Ceramic Primer Plus, Kuraray. Batch number: 1C0071	3-Methacryloxypropyl trimethoxysilane, MDP, ethanol
Dual cure resin cement	Panavia™ V5 Paste, Kuraray. Batch number: 1Q0188	Bis-GMA, TEGDMA, hydrophobic aromatic dimethacrylate, hydrophilic aliphatic dimethacrylate, initiators, accelerators, silanated barium glass filler, silanated fluoroalm glass filler, silinated barium aluminum glass filler, DL-Camphorquinone, pigments
Light cure resin cement	Panavia™ Veneer LC, Kuraray. Batch number: 2Q0002	Silanated spherical silica filler, UDMA, ytterbium trifluoride, TEGDMA, hydrophilic aliphatic dimethacrylate, hydrophilic amide monomer, accelerators, DL-Camphorquinone, pigments
Resin composite	Clearfil™ AP-X, Kuraray. Batch number: 170020	Bis-GMA, TEGDMA, silanated barium glass filler, silanated silica filler, silanated colloidal silica, DL-Camphorquinone.
Flowable composite	Clearfil Majesty™ Flow, Kuraray. Batch number: AM0024	Silanated barium glass filler, silanated colloidal silica, TEGDMA, hydrophobic aromatic dimethacrylate, DL-Camphorquinone.
Sandblast particle	Aluminium oxide	50 µm Al_2_O_3_

### Surface treatments

Before luting procedures, the polished side of ceramic, resin composite, and substrate discs received a surface treatment, as follows:

*Lithium disilicate:* The discs were pretreated as recommended by the manufacturer. The adherent surface was etched with 5% Hydrofluoric acid (HF) for 20 s. followed by thorough rinsing with running water for 30 s. Then, a silane coupling agent (Ceramic Primer Plus) was applied until the entire surface was coated.

*Resin composite:* The adherent surface received a surface treatment before luting, according to the manufacturer’s instructions. Each disc was subjected to 10 s of sandblasting (Harnissch + Rieth, P-G400) at 1.0 - 1.5 bar pressure with 50 μm aluminum oxide particles at a 10 mm distance. Subsequently, the discs were cleaned in an ultrasonic unit with 70% ethanol, rinsed with water spray, and dried with oil-free air. Afterward, a primer (PANAVIA™ V5 Tooth Primer, Kuraray) was scrubbed on the surface for 20 s. Subsequently, the primer was dispersed for 10 s with oil-/moisture-free compressed air.

*Dentin analog:* The bonding surface of the epoxy discs was etched with 10% HF for 60 s (Tribst et al., 2019), thoroughly rinsed with air-water spray for 30 s, and air-dried. Thereafter, the discs were pretreated equivalent to regular tooth dentin. A layer of primer (Panavia V5 tooth primer) was actively applied on the surface for 20 s. The prepared surface was thoroughly dried using a mild oil-free airflow.

### Luting procedures

The restorative discs were cemented on their respective substrate disc directly after the surface treatment described above. The luting took place according to each investigated luting procedure as follows:

*Dual-curing resin cement (dc)*: The dual-curing resin cement Panavia™ V5 was applied according to the manufacturer’s instructions in the center of the restoration surface that was bonded to the analog dentin disc. Subsequently, a standard load of 500 g was applied on the assembly on the occlusal surface (restoration side) promoting uniform cement spreading. The excess cement was removed using a microbrush and then, the set was light cured (high intensity of 1000 mW/cm^2^; wavelength ranging from 395 to 480 nm, Bluephase, Ivoclar) for 10 s at three points of the bonded surface followed by 10 s on the occlusal surface.

*Light-curing resin cement (lc)*: The protocol for the light-curing resin cement Panavia™ Veneer LC was the same as stated above for the dual-curing resin cement.

*Pre-heated resin composite (prc)*: A small amount of the resin composite (Clearfil™ AP-X) was inserted into a plastic dish and put in an oven with an internal temperature of 68 °C (Lohbauer et al., 2009; El-Deeb et al., 2015) for 5 min. Subsequently, the resin composite was immediately applied on the bonding restoration surface, and the set was placed under the 500 g load. The next steps followed the same protocol described for the resin cements.

*Pre-heated restoration and pre-heated resin composite (pr + prc)*: The protocol for the *pr + prc* group was performed in the same way as the *prc* group. The difference in this group was that together with the resin composite, the restoration disc was also heated in the oven. After, the luting was immediately performed following the steps described above.

*High-filled flowable resin composite (frc)*: The discs cemented with Clearfil Majesty™ followed the same luting procedure described above for resin cement.

All luted specimens were stored for 24 h in distilled water at 37 °C, before the flexural strength test. In total, 400 discs were divided into 20 groups, according to the restorative material (2 levels), restoration thickness (2 levels), and luting procedure (5 levels), totaling 20 specimens per group.

### Biaxial Flexural Strength

The specimens were mounted on a ring (inner Ø = 6.5 mm, outer Ø = 10 mm, thickness = 1.5 mm) to ensure flexibility in the specimen (de Kok et al., 2017). The restoration surface was placed perpendicular to the load source (compressive side). A universal testing machine (INSTRON 6022) with a static load cell of 10kN, was set with a linear downward speed of 0.5 mm/min for testing. For this study, initial cracking was considered as failure mode (Tribst et al., 2022). The load-to-failure (N) was recorded when the initial cracking sound during testing was detected. The mean load to failure ± standard deviation data of each group was considered to perform simulations and to calculate the results in MPa per specimen. Therefore, individual simulations were performed considering the failure load (in N) obtained from the in vitro test. For that, the biaxial flexural strength at fracture (maximum tensile stress) in the center of the specimen was calculated using the mechanical simulation of the in vitro setup (Figure 1).

Biaxial flexural strength refers to the maximum stress a material can withstand when subjected to bending forces applied in two perpendicular directions (usually along two orthogonal axes). However, the Biaxial Flexural Strength is a calculated or estimated value based on correlations between the material’s uniaxial strength and its expected behavior under biaxial loading. In summary, the size, shape, and aspect ratio of the specimen can influence its behavior under bending loads (Kanit et al., 2006). Since the present study considered a bonded specimen, the measured property should be considered the calculated Biaxial Flexural Strength based on Maximum Principal Stress, showing the effect of different luting procedures on the material’s strength.

To calculate the Maximum Principal Stress, a three-dimensional (3D) Finite Element Analysis was performed considering the dimension of the in vitro specimen, with a standardized cement layer thickness of 100µm. The geometries were designed using a computer-aided software program (Rhinoceros, version 5.0 SR8, McNeel North America) and then, imported in Standard for the Exchange of Product (STEP) data format to the analysis software (ANSYS 17.2, ANSYS Inc.). After a convergence test (10%), tetrahedral elements formed the mesh, and the mechanical properties of each material were considered with isotropic behavior (Table 2).

**Table 2.** Mechanical properties of simulated materials.

Material	Elastic modulus (GPa)	Poisson ratio	Reference
CAD/CAM resin composite	10.2	0.30**	Marchesi et al., 2021
Lithium disilicate	95.0	0.25	Dal Piva et al., 2018
Dual cement	2.0*	0.30**	–
Light-curing resin cement	1.7*	0.30**	–
Resin composite	16.8	0.26	Park et al., 2017
High-filled flowable resin composite	8.8	0.30**	Sumino et al., 2013
Dentin analog	18.0	0.30	Chen et al., 2014

* Calculated by the authors using the 3-point bending test described by de Kok et al. (2015) for 5 specimens from each evaluated cement.

** A Poisson ratio of 0.30 was assumed.

### Scanning Electron Microscopy (SEM)

The specimens from all groups were cross-sectioned and the failure was analyzed using SEM (EVO LS15, Zeiss, Oberkochen, Germany) to determine its origin and direction of crack propagation. For that, the specimens were sputter-coated with gold for 130 s at 15 mA and inspected with magnifications of 100x and 500x. The cement layer thickness was also measured for qualitative comparison between groups.

### Statistical analysis

Load-to-failure descriptive data were calculated to be further used in the Finite Element Analysis. Therefore, no statistical analysis was applied to these values. All results were tested on normality using the Ryan-Joiner Normality test (similar to Shapiro-Wilk). Data were then analyzed using 3- and 2-way ANOVA (Analysis of Variance) followed by the post-hoc Tukey test. The significance level (α) in the study was set at 5%. P-values below this level were considered statistically significant.

## Results

### Calculated Biaxial Flexural Strength

After proving data normality, 3-way ANOVA revealed that restorative material, restoration thickness, and luting procedure each influenced the flexural strength of the bonded restorations (p values <0.001), as did their interactions (Table 3).

**Table 3.** Three-Way Analysis of Variance (ANOVA) for the flexural strength: Comparison of restorative material, restoration thickness, and luting procedure.

Source	DF	Adj SS	Adj MS	F-Value	P-Value
Material	1	23145176	23145176	5608.47	0.000
Thickness	1	23310	23310	5.65	0.018
Protocol	4	1049804	262451	63.60	0.000
Material*Thickness	1	158855	158855	38.49	0.000
Material*Protocol	4	150701	37675	9.13	0.000
Thickness*Protocol	4	79418	19855	4.81	0.001
Material*Thickness*Protocol	4	52265	13066	3.17	0.014
Error	380	1568193	4127		
Total	399	26227723			

Considering pooled values for restorative material factor, lithium disilicate restorations showed higher mean flexural strength (570 MPa) than resin composite (90 MPa), and thicker restorations (338 MPa) were stronger than the thinner ones (323 MPa). Regarding the luting procedure, *lc* showed the highest mean value (409 MPa)^A^, compared to *dc* (362 MPa)^B^*, pc + prc* (317 MPa)^C^, *prc* (304 MPa)^C^, and followed by *frc* (259 MPa)^D^. Table 4 summarizes the data variation according to the groups as well as the grouping test according to the interaction of factors.

**Table 4.** Flexural strength (MPa)* mean values ± standard deviation (sd) for each restorative material according to the evaluated thickness. Mean cement layer thickness at cross-sectioned specimen (µm).

Luting Procedure	Restoration thickness (mm)	Lithium Disilicate			Resin Composite		
		load to fail (N)	Calculated Flexural Strength (MPa)	Cement layer (µm)	load to fail (N)	Calculated Flexural Strength (MPa)	Cement layer (µm)
Dual-curing Resin cement – *dc*	0.5	286 ± 50^K^	622 ± 89^B^	168.5	623 ± 110^IJ^	138 ± 2^FG^	49.2
Light-curing Resin cement – *lc*		277 ± 34^K^	628 ± 68^B^	50.1	1406 ± 163^EF^	165 ± 4^F^	121.9
Pre-heated resin composite – *prc*		394 ± 68^JK^	540 ± 82^CD^	145.7	936 ± 150^G^	75 ± 1^GHI^	52.1
Pre-heated crown + pre-heated resin composite – *pc + prc*		440 ± 117^IJK^	594 ± 138^BC^	204.5	927 ± 182^GH^	75 ± 1^GHI^	84.9
High-filled flow composite - *frc*		229 ± 115^K^	398 ± 184^E^	137.1	684 ± 178^HI^	57 ± 5^HI^	70.3
Dual-curing Resin cement – *dc*	1.5	1500 ± 190^DE^	612 ± 67^BC^	133.3	1231 ± 276^F^	80 ± 3^GHI^	128.3
Light-curing Resin cement – *lc*		1762 ± 98^C^	717 ± 34^A^	132.1	3119 ± 642^A^	121 ± 13^FGH^	62.7
Pre-heated resin composite – *prc*		1727 ± 193^CD^	551 ± 51^BC^	88.5	1887 ± 116^BC^	66 ± 7^GHI^	74.4
Pre-heated crown + pre-heated resin composite – *pc + prc*		1818 ± 118^BC^	575 ± 48^BC^	127.2	2067 ± 399^B^	73 ± 18^GHI^	64.4
High-filled flow composite - *frc*		1495 ± 192^DE^	519 ± 57^D^	100.2	1295 ± 214^EF^	48 ± 7^I^	76.7

* Different capital letters correspond to significant differences between luting procedures for each restorative material thickness. Similar small letters mean no significant difference between luting procedures for each material.

**Figure 2.**
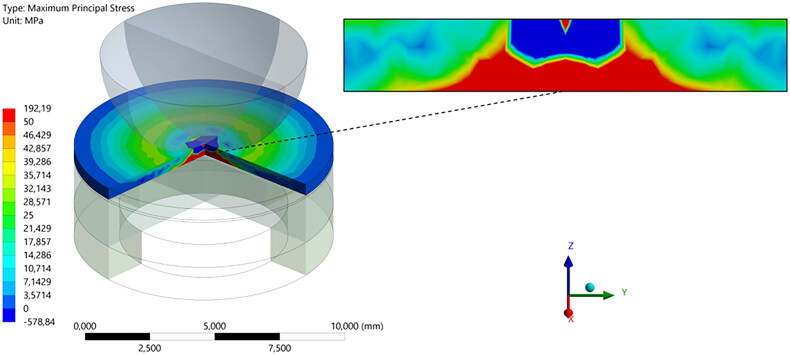
Representative highest tensile stresses (Maximum Principal Stress) in the restoration after processing of results.

**Figure 3.**
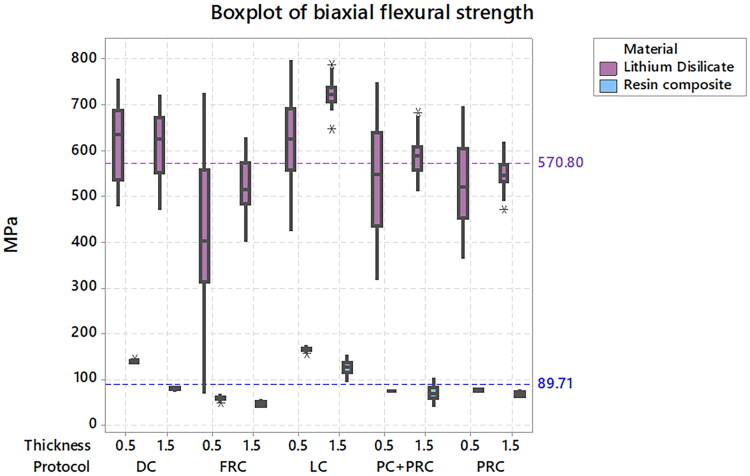
Boxplot of the biaxial flexural strength according to each evaluated condition. The reference gridlines show the average strength per restorative material.

The load-to-failure descriptive data used in the Finite Element Analysis is also presented in Table 4. Figure 2 is an illustration of a representative highest tensile stress (Maximum Principal Stress) in the restoration model after processing of results. The image shows the stress concentration on the bottom of the specimen corresponding to the tensile stress concentration. Based on the biaxial flexural strength calculated using FEA, Figure 3 presents a boxplot according to each evaluated condition as well as the average strength per restorative material regardless of the luting procedure.

To assert the effect of force, one-way ANOVA was performed and revealed that the group’s procedure influenced the initial fracture load of bonded restorations (p values <0.001) (Table 3).

### Scanning Electron Microscopy (SEM)

**Figure 4.**
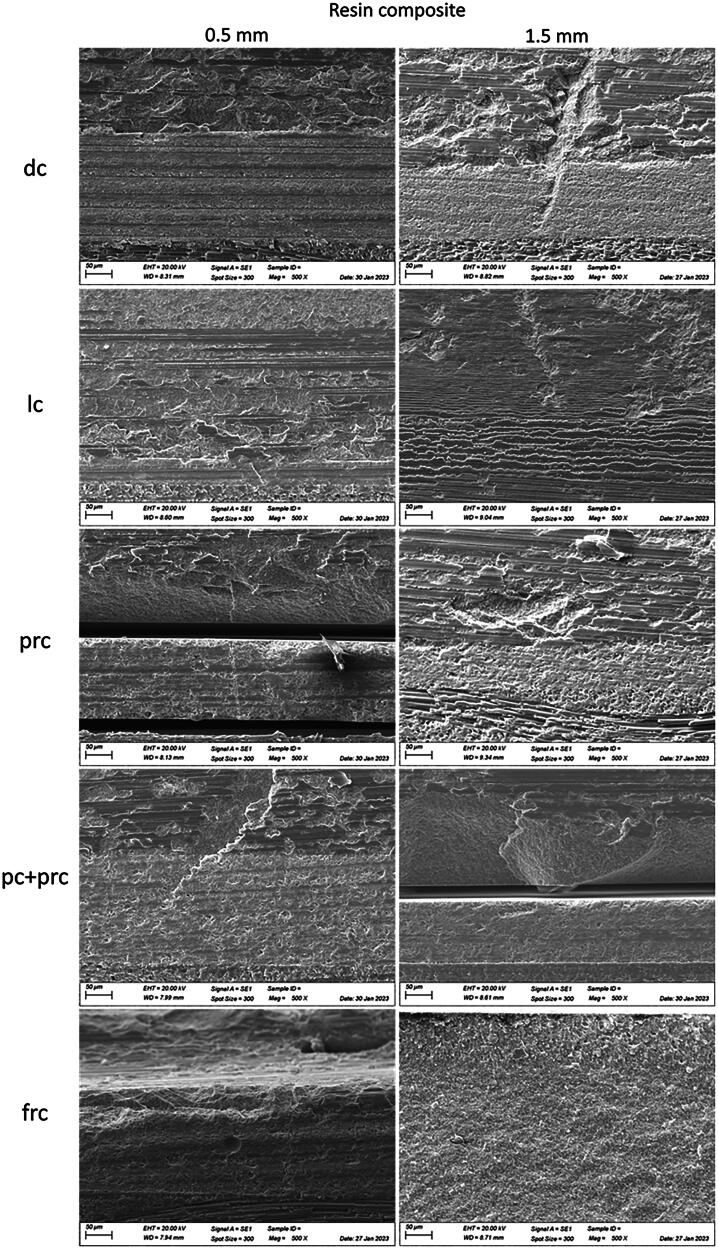
Failure origin of failed cemented resin composite discs (thickness 0.5 and 1.5 mm) luted according to different luting procedures (500x).

**Figure 5.**
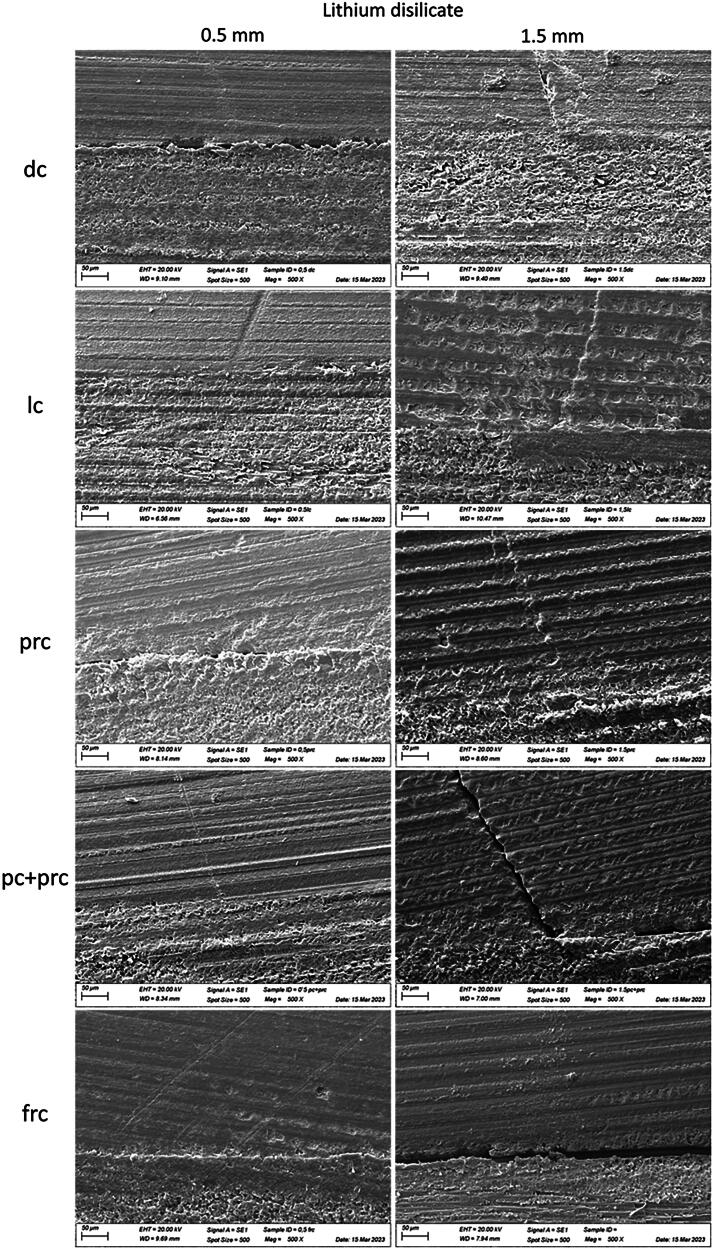
Failure origin of failed cemented lithium disilicate discs (thickness 0.5 and 1.5 mm), luted according to different luting procedures (500x).

SEM revealed that all failures originated on the cement layer and propagated as radial cracks toward the restorative material. This finding can be observed in Figures 4 and 5, which present representative failed specimens analysis of the different luting procedures used for the resin composite discs and lithium disilicate with a thickness of 0.5 mm and 1.5 mm, respectively. The images display the failure site with 100x and 500x magnifications.

## Discussion

This study aimed to investigate the influence of luting procedures and restoration thickness on the biaxial flexural strength of cemented CAD/CAM lithium disilicate and resin composite. To determine the flexural strength of tri-layer specimens, first, they were submitted to compressive test to obtain the fracture load values. The load in Newtons and standard deviation were considered in a finite element analysis (FEA) to obtain the flexural strength value at failure (in MPa) per specimen; therefore, showing the maximum center stress during the failure of adhesively cemented discs, as previously described (Tribst et al., 2020).

The biaxial flexural strength is a property used to evaluate the mechanical strength of a material. It specifically measures the material’s ability to withstand bending forces applied in two perpendicular directions. Biaxial flexural strength is often used to assess the suitability of materials for applications where they may be subjected to complex loading conditions or where strength and resistance to fracture are crucial factors (Ban and Anusavice 1990, Beyabanaki et al., 2022). When a restoration is cemented, the mechanical properties of the cement layer can affect the overall behavior of the restoration (de Kok et al., 2017). This intermediate flexible layer can contribute to the load-bearing capacity, stress distribution, and fracture resistance of the restoration, providing additional support to the underlying tooth structure (Tribst et al., 2019). Furthermore, the bonding between the restoration and the tooth structure can enhance the overall mechanical performance of the restoration (de Kok et al., 2017, Pucci et al., 2022). On the other hand, non-bonded restorations may have different stress distribution patterns and mechanical behavior compared to cemented restorations (de Kok et al., 2017).

Resin composite bonded to the dentin analog presented lower flexural strength compared to lithium disilicate, accepting the first hypothesis. Overall, CAD/CAM resin composite can provide a higher fracture load than CAD/CAM lithium disilicate (Table 4) due to its material properties, processing, and design flexibility. Resin composite can be cured under high pressure and temperature, which can improve its strength and reduce its porosity. However, in terms of flexural strength, lithium disilicate is a stiffer material with a 9 times higher elastic modulus compared to the evaluated composite. Therefore, lithium disilicate has the ability to concentrate more stresses under loading; thus, it presents higher strength against failure (Dal Piva et al., 2018). This behavior was proportional to the comparison between thicker and thinner restorations.

For both restorative materials, thickness affected the flexural strength. This finding was expected since this mechanical property is not fully inherent to the material and the specimen dimensions of the tri-layer setup can partially affect it. Therefore, the second hypothesis was accepted. It can be explained by thicker restorations having a higher ability to resist bending and deformation when a load is applied perpendicular to its surface, mainly due to their greater volume which allows for a more homogeneous distribution of stresses (Dal Piva et al., 2021). When a force is applied to a thin restoration, such as a veneer, the material can easily bend or deform. The values found in the present investigations (1500 N) are in agreement with the finding of a previous report that evaluated also evaluated 1.5 mm thick lithium disilicate discs (1613 N) bonded with dual-curing cement on dentin analog under similar conditions (Chen et al., 2014). On the other hand, thicker restorations, such as a crown, have a greater cross-sectional area and are therefore able to resist bending and deformation to a greater extent. This means that the load is distributed over a larger area, reducing the stress concentration at any given point and increasing the overall strength of the restoration. However, in this study, the difference between 0.5 and 1.5 mm thickness was evident only in some groups. The finding that increased thickness did not always lead to higher flexural strength may be explained by the fact that the restorations were bonded; similar to a previous report (de Kok P, et al., 2017), it is possible that bonded restorations present high flexural strength than non-bonded ones, showing that the luting agent and polymerization shrinkage could also affect the mechanical response of restorations. According to the manufacturer, the average biaxial flexural strength of lithium disilicate is 500 MPa (IPS website) while for resin composite (Ivoclar) it is approximately 274 MPa. This study found that, when bonded, lithium disilicate had similar or even higher flexural strength than that stated by the manufacturer (approximately 25%) when resin cement (light- or dual-curing) was used for thinner restorations and approximately 40% higher when light-curing resin cement was used for thicker restorations. However, when thinner restorations were cemented with high-filled flowable composite, the average flexural strength was 20.4% lower than the manufacturer’s information. Considering the data from a previous study, the flexural strength of lithium disilicate was 278 MPa after polishing in non-bonded situations (Fraga et al., 2017), sugesting that all luting procedures could have increased the material’s strength.

On the other hand, for resin composite, no group showed flexural strength values as high as the value reported by the manufacturer, with the light-curing resin cement being the most promising option for both evaluated thicknesses. Therefore, it can be suggested that a brittle material (lithium disilicate), when cemented, promotes a stronger setup of crown/cement/dentin while a cemented flexible material (resin composite) promotes a weaker setup in comparison with the intrinsic properties of the restorative material itself. Another important factor was that the load-to-failure test was suspended when the first crack appeared in the specimen, a feature that can be generated by failures in the cement layer. The presence of cracks in the specimen corroborates with the resin composite results since it was reported that this restorative material allows a higher stress concentration in the cement layer when loaded (Dal Piva et al., 2018; Tribst et al., 2019).

The MPa value corresponds to a measure of the maximum stress that a material can withstand before it bends or breaks under a bending test. In general, a material with a high flexural strength will be able to withstand greater bending loads without breaking or deforming, making it more suitable for applications that require high strength and rigidity (de Jager et al., 2021). However, the relationship between thickness and flexural strength is not always straightforward and can depend on other factors, such as composition, microstructure, processing history, and defect population. Additionally, in some cases, increasing the thickness of a material may lead to a decrease in its bending strength due to factors such as residual stresses or more defects introduced during processing (Thomason, 2001).

Considering both materials and thicknesses together, lithium disilicate showed the highest mean flexural strength. In addition, luting procedures promoted different flexural strength values considering each material and thickness separately, accepting the third hypothesis. For lithium disilicate at 0.5 mm, dual-curing cement and pre-heated crown + pre-heated resin composite were also similar to light-curing cement. Therefore, the promising behavior of heated resin composites must be further investigated to cement lithium disilicate restorations. This could be associated with the resin composite results that showed a slight reduction in the strength values when the thickness was increased.

In clinical practice, the temperature from the heated resin composite can easily drop when it is inserted in the oral cavity (Rueggeberg et al., 2010), suggesting that the heating may not produce the increase in physical properties demonstrated by in vitro studies (Rueggeberg et al., 2010; Vandewalle et al., 2010). Previous authors also mentioned that heating the composite improves the handling, and no loss of physical properties has been found with the use of flowable resin composite (Vandewalle et al., 2010). Thus, a temperature drop subdues the adaptation of the composite to the cavity walls, but it does not affect the flexural strength (Froes-Salgado, 2010). In the present study, results showed that high-filled flow composite presented the lowest mean values. The outcome of this study regarding the high-filled flowable resin composite can be explained when taking its filler content weight and size (81 wt%, 62 vol%) into account. Studies suggest that there is a correlation between physical and mechanical properties and filler content (weight and size) in resin composites. A study by Tanimoto et al., (2006), showed that an increase in filler particle size caused an increase in stress concentration and a decrease in flexural strength. Regarding dual-curing cement, filler contents (61 wt.%, 38 vol.%) are relatively low compared to the other luting agents used in this study. A study by Randolph et al., (2016), showed that there is a correlation between filler content percentages and mechanical properties, including flexural strength. The study showed a direct proportionality since the higher the filler content, the higher the flexural strength. Also, a study by Barbon et al., (2019), showed that increased filler content resulted in higher flexural strength.

According to previous reports, the use of pre-heated resin composite as a luting agent provides a slight improvement in the mechanical properties of indirect restorations (Froes-Salgado 2010). However, a systematic review with meta-analysis showed that using pre-heated composite presents minor improvements in mechanical properties and it is also associated with an unacceptably high film thickness (Barbon et al., 2022). These findings are not supported by this study due to the observed intermediate behavior of heated composites and cement layer thicknesses. The cement layer thickness, or film thickness, was evaluated since a relation of higher thickness and lower bond strength has been suggested for bonding procedures. A thicker layer is more prone to the incorporation of defect which can initiate failures. However, the results on film layer thickness in the present study must be evaluated with caution as these results were evaluated only on representative specimens. In addition, low viscous material presents higher flow capability and, therefore can be spread more homogenously and faster on the adhesive surface (Dapieve et al. 2023) with less or no difficulty, facilitating the luting procedure. In the present study, the resin composite was heated to 68 °C to allow comparison with previous studies (Sampaio et al. 2017; Sartori et al. 2016).

In summary, heated resin composite luting is an option that will promote flexural strength similar to that of conventional dual-curing resin cement, but with more clinical steps. Since clinicians aim for clinical success, procedures that are practical or less prone to errors are preferable, and the increase in the number of clinical steps involved when using the heated resin composite technique seems to be an obstacle for widespread use. Nevertheless, a prospective clinical evaluation of 765 lithium disilicate partial posterior restorations cemented using heated resin composite in conjunction with immediate dentin sealing showed excellent prognosis after 5 years (Van den Breemer et al. 2021). Despite the reported findings, the authors see a need for more investigations to confirm whether the benefits justify the more extensive procedure.

It is important to mention that pre-heated resin composites used for luting also offer an enormous variety of colors compared to resin cements, which typically have a limited range of shades. Additionally, many resin cement manufacturers provide the option to perform a restoration test using try-in pastes that match the color of the resin cement after polymerization and hydration. However, those factors have been not evaluated in this investigation. The limitations of this study consisted in the use of only one temperature to heat the composite and in a limited number of resin cements and composites. In addition, no aging procedure was applied to evaluate the long-term performance of the restorations.

## Conclusion

CAD/CAM resin composite restorations with a thickness of 0.5 or 1.5 mm should be cemented using light-curing resin cement to enhance flexural strength. Thinner restorations made of CAD/CAM lithium disilicate can be luted with any of the investigated luting procedures. For thicker lithium disilicate restorations, options such as dual-curing cement and pre-heated resin composite, in combination or not with a pre-heated restoration should be considered.

## Clinical significance

Using light-curing resin cement during the luting procedure promotes high mean flexural strength to CAD/CAM restorations made of lithium disilicate and resin composite. However, it is important to consider other factors such as aesthetics and the experience of the operator when choosing the luting agent for lithium disilicate restorations.

## Data Availability

Data will be available as requested.
